# Depression and suicidal behavior in adolescents: a multi-informant and multi-methods approach to diagnostic classification

**DOI:** 10.3389/fpsyg.2014.00766

**Published:** 2014-07-17

**Authors:** Andrew J. Lewis, Melanie D. Bertino, Catherine M. Bailey, Joanna Skewes, Dan I. Lubman, John W. Toumbourou

**Affiliations:** ^1^School of Psychology, Deakin UniversityMelbourne, VIC, Australia; ^2^Eastern Health Clinical School, Turning Point, Monash UniversityMelbourne, VIC, Australia

**Keywords:** adolescent depression, suicide, psychiatric diagnosis, classification, parent-child relations

## Abstract

**Background:** Informant discrepancies have been reported between parent and adolescent measures of depressive disorders and suicidality. We aimed to examine the concordance between adolescent and parent ratings of depressive disorder using both clinical interview and questionnaire measures and assess multi-informant and multi-method approaches to classification.

**Method:** Within the context of assessment of eligibility for a randomized clinical trial, 50 parent–adolescent pairs (mean age of adolescents = 15.0 years) were interviewed separately with a structured diagnostic interview for depression, the KID-SCID. Adolescent self-report and parent-report versions of the Strengths and Difficulties Questionnaire, the Short Mood and Feelings Questionnaire and the Depressive Experiences Questionnaire were also administered. We examined the diagnostic concordance rates of the parent vs. adolescent structured interview methods and the prediction of adolescent diagnosis via questionnaire methods.

**Results:** Parent proxy reporting of adolescent depression and suicidal thoughts and behavior is not strongly concordant with adolescent report. Adolescent self-reported symptoms on depression scales provide a more accurate report of diagnosable adolescent depression than parent proxy reports of adolescent depressive symptoms. Adolescent self-report measures can be combined to improve the accuracy of classification. Parents tend to over report their adolescent’s depressive symptoms while under reporting their suicidal thoughts and behavior.

**Conclusion:** Parent proxy report is clearly less reliable than the adolescent’s own report of their symptoms and subjective experiences, and could be considered inaccurate for research purposes. While parent report would still be sought clinically where an adolescent refuses to provide information, our findings suggest that parent reporting of adolescent suicidality should be interpreted with caution.

## INTRODUCTION

The classification of psychiatric disorders is an area of controversy and particularly challenging in child and adolescent mental health. Accurate diagnostic classification can be used to predict benefit from treatment, predict duration, or severity of disorder and is central to etiological research. Researchers conducting clinical trials need to make use of valid measures for diagnostic classification to examine both eligibility and intervention outcomes (Lewis et al., 2012b). Eligibility criteria for entry to a clinical trial are ideally based on a valid and reliable diagnostic measure in order to ensure that any claim that the intervention is effective can be used as an evidentiary recommendation for all patients presenting with the same psychiatric disorder. Clinically significant effects of a given intervention are assumed to reflect therapeutic change from a state of diagnosable disorder to a state of remission. The underlying assumption is that psychiatric disorder can be classified in a robust manner. Many clinical researchers also operationalize clinical change in terms of reductions in symptom levels and argue that such an approach may be more sensitive to identifying treatment effects. Often the preferred outcome measures for intervention studies depends on the researcher’s conceptualization of disorder as either a categorical or a continuum of levels of impairment. However, there is much to be gained from the adoption of a multi-methods approach derived from multiple informants within clinical research designs, particularly in the assessment of children and adolescents.

In this paper, we examine a multi-informant and multi-method approach to diagnosing depressive disorders in adolescents. We draw on data from a clinical trial (The Family Options Study) for adolescent depression in which adolescents (12–18 years of age) needed to meet diagnostic criteria on a semi-structured interview for a depressive disorder for entry to the trial (Lewis et al., 2013). This study trialed two forms of family based intervention over an 8 week period and recruited 50 families. The study also collected self-report symptom count measures of depression, measures of depressive personality features, and also a matched set of measures from the adolescent’s parent about the adolescent’s depression. This rich clinical dataset posed many important questions about the optimal use of multiple sources of information and different diagnostic methods within the context of a clinical trial (Eid and Diener, 2006).

Establishing valid diagnostic classification for the purposes of determining eligibility for a clinical trial, or to determine response to treatment, encounters many specific challenges when conducting mental health research with children and adolescents (Petersen et al., 1993; Oetzel and Scherer, 2003; Achenbach, 2005; Mash and Hunsley, 2005). Children below a certain age may not have the capacity to reliably self-report on constructs relevant to mental disorder. To address some of these issues, researchers typically supplement the assessment of children and adolescents with parent proxy reports. This is commonly done with parents via a questionnaire, and many instruments such as the Child Behavior Checklist and the Strengths and Difficulties Questionnaire (SDQ) have validated parent and adolescent report versions. However, parent proxy reporting of depressive disorder is less frequently attempted using a diagnostic interview. In a multi-informant model, there are three sources of variance: derived from the trait itself, variance derived from the source of information, and measurement error (Burns and Hayes, 2006). Source error is pervasive in psychiatric classification since the setting in which information is gathered and the profession of those gathering it has been shown to influence diagnosis of a number of disorders (Commons Treloar and Lewis, 2009). As applied to child and adolescent mental disorders, variance derived from the source may arise from not being accurate reporters, their own over or under reporting of their own symptoms, or they may be difficult to engage in a diagnostic interview process.

The diagnosis of depression is particularly significant given its high population prevalence and increasing incidence across adolescence (Kessler et al., 2011; Thapar et al., 2012). Drawing information from multiple sources is a standard part of clinical assessment and practice parameters for adolescent depression recommend direct interviews with both the child and parents and that an adolescent should also be interviewed alone. Guidelines also recommend that other informants provide information which may include teachers, general practitioner, community service professionals, and in some cases peers (AACAP, 2007). Information from multiple sources is generally thought to address common issues in assessing adolescents for depressive and suicidal symptoms, such as the high stigma of these conditions, accompanied by social withdrawal and feelings of guilt that may inhibit accurate reporting. However, Achenbach’s et al. (1987) meta-analysis of 119 studies with multi-informant ratings on child behavior problems suggested average correlations of 0.59 between mother and father, 0.27 between parent and teacher, 0.25 between parent and child, and 0.20 between teacher and child (Achenbach’s et al., 1987; Rowe and Kandel, 1997). It remains unclear which combination of measures and which combination of reporters is optimal and how these are best applied within a clinical research context.

Diagnostic interviews are the preferred method used to establish clinical diagnosis given these approaches are designed to investigate the specific diagnostic features listed in the Diagnostic and Statistical Manual of Mental Disorders. Several standardized structured and semi-structured interviews are available for the evaluation of mental disorders in children older than 7 years and some measures have been adapted for assessment of preschool aged children (Luby et al., 2003). These interviews are typically either fully structured or semi-structured to allow assessors scope for probing or follow up questions, which are designed to further investigate the presence, duration or intensity of a given symptom and typically require training to be used accurately. The most commonly used clinical interview measures used in child and adolescent mental health are: the Schedule for Affective Disorders and Schizophrenia for School-age Children (K-SADS), The Child Assessment Scale, The Anxiety Disorders Interview Schedule for Children, The Diagnostic Interview for Children and Adolescents, and the Diagnostic Interview Schedule for Children (Grills and Ollendick, 2002). In this paper we report on the use of the Structured Clinical Interview for DSM-IV Childhood Diagnoses (KID-SCID), a child and adolescent version of the well-established adult SCID.

However, notwithstanding the recommendation for multi-informant approaches, clinical practice guidelines also warn that measures of adolescent depression have low parent-child agreement (AACAP, 2007). Parent’s proxy reports of adolescent symptoms are expected to be non-identical because of the influence of parent’s own mental state, the parent-adolescent relationship and parent personality functioning (Bertino et al., 2012). So too teacher reports may be biased by insufficient knowledge of a given child or observations derived from different behavior in a different context. For this reason, multi-informant approaches, while generally preferred clinically in child and adolescent mental health, often show discrepancies in the classification of disorders between different informants be they parents, children or teachers (De Los Reyes and Kazdin, 2005; Sznajder et al., 2013). For example, a study by Lauth et al. (2010) using a sample obtained from an adolescent inpatient setting reported poor parent–adolescent agreement for most symptoms, but particularly for depressive symptomatology and reports of suicidal ideation. The inconsistency was attributed to both over and under reporting by parents (Lauth et al., 2010). Similarly, in another study, Dolle et al. (2012) reported low agreement between child and parent diagnostic interviews. De Los Reyes and Kazdin (2005) reviewed studies seeking to identify informant characteristics that influence such discrepancies and found that parent proxy report on adolescent mental health symptoms is often biased. Kiss et al. (2007) investigated the predictors of discrepant reports by mothers and their adolescents in a Hungarian sample and found that mothers reported higher symptoms for their sons than their daughters, while female adolescents self-reported higher levels of symptoms than boys. In this study, maternal depression was also found to be a predictor of reporting higher levels of symptoms in children. Kiss et al. (2007) also found that agreement between the mother and the child increased as children aged. Finally, in a study by Ko et al. (2004) adolescents were found to report higher rates of disorder than parents and parents were more likely than adolescents to report that disorders were causing functional impairment.

In the present study we hypothesized, firstly, that single informant models will show modest concordance between parent and adolescent reports of adolescent’s depressive and suicidal symptoms. Our second hypothesis was that adolescent questionnaire measures of depressive symptoms would more accurately reflect adolescent diagnosis than parent reports on adolescent symptoms. Our third hypothesis was that in a multi-measures approach tested within a multivariate model, adolescent self-reports would have relatively greater importance than parent proxy report in distinguishing amongst adolescents with current major depressive episodes (MDE) compared to those without MDE. In order to compare adolescent and parent self report in a multivariate model we undertook discriminant functional analysis.

## MATERIALS AND METHODS

### STUDY DESIGN AND SAMPLE

The Family Options trial aimed to measure the relative efficacy of two intervention programs aimed at treating adolescents who present with a unipolar mood disorder, here defined as major or minor adolescent depression (see Lewis et al., 2013; for full study protocol). With both treatments, families received eight, 2 h sessions of treatment delivered weekly. The trial was run over a number of sites in Metropolitan Melbourne and the regional Victorian city of Geelong. The families were primarily recruited from the intake service of a large government run mental health service in the eastern region of Melbourne (Eastern Health’s Child and Youth Mental Health Service, CYMHS), as well as community referrals from schools and community based health and mental health services.

The primary outcome measure of the study was the rate of remission of the depressive disorder, using modules from the KID-SCID (Hien et al., 2004). Secondary outcome measures were reductions in adolescent depressive symptoms as reported by both the parent and the adolescent.

Analysis for this study draws on the data from the baseline assessment of participants recruited into the Family Options trial. The sample consisted of 50 parents and their adolescents of whom 14 were male (26.0%) and 36 female (72.0%). Age ranged from 12 to 19 years, with an average age of 15.0 years (SD = 1.57). Assessments of the parent and adolescent were done within 1 week of each other, and are therefore considered to be observing the same behavior over the same time period. The KID-SCID was conducted with adolescents with sufficient modules to establish a diagnosis of Major Depressive Disorder (MDD), however, the parent version of the KID-SCID used only the depressive episodes module rather than all modules necessary to establish the presence of a mood disorders. Hence, only the MDE module of the adolescent KID-SCID was utilized in this study, so that the adolescent and parent KID-SCID diagnoses were directly comparable. The study protocol excluded adolescents who met the diagnostic criteria for Psychotic Disorders, Autism, and Major Substance Abuse. The primary inclusion criterion was meeting a Diagnosis of Major or Minor Depression or Dysthymia on the KID-SCID.

### MEASURES

#### Diagnostic interview for depressive disorders

The KID-SCID is a semi-structured instrument designed to generate childhood DSM 4th Edition (DSM-IV; American Psychiatric Association, 1994) diagnoses for clinical research studies. This instrument is modeled on the extensively used adult version (SCID). The modular nature of the KID-SCID permits users to select modules relevant to their specific research protocol. For the purposes of this paper, the MDE module was analyzed as completed by both the adolescent and their parent. The KID-SCID modules were conducted via telephone with adolescent and parents to determine eligibility and to assess the primary study hypothesis. The selected module was tailored for use in the randomized controlled trial (RCT) such that two versions were created: one for completion with the adolescent, and another for completion with a parent/caregiver, with personal pronouns changed to the third person.

#### Adolescent suicidality

Within the KID-SCID MDE module there is a specific question about suicidality (with follow up prompts). The participant is asked to focus on the worst 2 weeks in the past month when the most severe lowered mood, irritable mood and/or anhedonia were experienced. Adolescent participants were then asked: “During that (2 week period)...were things so bad that you were thinking a lot about death, or that you would be better off dead?” “What about thinking of hurting yourself?” (IF YES) “Did you do anything to hurt yourself?” The parent was also asked the same item about their adolescent offspring with modified wording, as follows: “During that (2 week period)...were things so bad that your young person (use their name) told you or others they were thinking a lot about death, or that they would be better off dead?” “What about thinking of hurting themselves?” (IF YES) “Did they do anything to hurt themselves?” Responses to these questions were then evaluated with regard to whether they meet the DSM-IV criteria of “recurrent thoughts of death (not just fear of dying), recurrent suicidal ideation without a specific plan, or a suicide attempt or a specific plan for committing suicide” and coded as present or not present.

### DEPRESSIVE SYMPTOM QUESTIONNAIRES

#### Strengths and Difficulties Questionnaire

The SDQ consists of 25 items (Goodman et al., 1998), and has been validated with both parent-report and adolescent self-report versions (Goodman and Scott, 1999; Goodman, 2001; Goodman et al., 2004). The items load onto five subscales relating to adolescent mental health, including emotional symptoms (ES), conduct problems, hyperactivity/inattention, peer relationship problems, and pro-social behavior. The SDQ has already been developed and validated with both parent-report and adolescent self-report versions (Goodman and Scott, 1999; Goodman, 2001; Goodman et al., 2004). For this study, we only used the ES subscale for parent and adolescent self-report, consisting of five items, with higher scores indicating more ES. Cut points for determining sensitivity and specificity analysis were taken from Mellor (2005), and are normed for the Australian population. Cut points for self-report were 5+ for boys and 6+ for girls (ages 11–17 years) and for parent report were 5+ for boys and 5+ for girls (ages 11–17 years). According to Mellor (2005), scores on or above these cut points indicate that the participant would be classified into the clinical range for “high emotional symptoms” and would require further screening. Adequate internal consistency was found for the data used in this study (adolescent SDQ total alpha = 0.74; adolescent SDQ ES subscale alpha = 0.79; parent SDQ total alpha = 0.74; parent SDQ ES subscale alpha = 0.65).

#### Short Mood and Feelings Questionnaire

The Short Mood and Feelings Questionnaire (SMFQ) consists of 13 items, and is based on the 34-item Moods and Feelings Questionnaire. Higher scores reflect more depressive symptoms (Messer et al., 1995). The questionnaire was administered to both parents (reporting about adolescent) and adolescent (self-report), with wording for the parent version modified to the third person (“She or He”). A cut point of 11+ was used to indicate clinically significant levels of depressive symptoms (Messer et al., 1995). There is a considerable amount of research indicating that the SMFQ has good reliability and validity (Costello and Angold, 1988; Messer et al., 1995). Adequate internal consistency was found for the data used in this study (adolescent SMFQ alpha = 0.94; parent SMFQ alpha = 0.88).

#### Depressive Experiences Questionnaire for adolescents

The Depressive Experiences Questionnaire for adolescents (DEQ-A shortened) is a 20-item questionnaire designed to assess personality characteristics associated with depressive symptoms (Blatt et al., 1992), and was developed from the 66-item DEQ for adults (Blatt et al., 1976). Items were identified from the DEQ according to their loading onto two subscales; dependency and efficacy (Blatt et al., 1982, 1992, 1995). Since this measure is not specifically focussed on symptoms, it is not generally regarded as diagnostic and is not used with clinical cut points. Adequate internal consistency has been demonstrated of alpha in the range of 0.65 to 0.70 (Blatt et al., 1992). In our study internal consistency for the DEQ-A was reasonable for DEQ-A dependency subscale (alpha = 0.70) but the DEQ-A efficacy subscale was found to have alpha = 0.50. We persisted with the use of the dependency subscale since depression where high interpersonal dependency occurs in adolescence is of particular theoretical and clinical interest but note that interpretation needs to be undertaken with caution.

### DATA ANALYSIS

All analyses were performed using SPSS version 21 (SPSS Inc, Chicago, IL, USA). There was no missing data for the adolescent and parent KID-SCIDs because their completion was a requirement for entry to the trial. There was one case of missing data for an adolescent’s demographic variables, and 15 cases of missing data for the adolescent questionnaires, due to the adolescent having been screened with the KID-SCID as not being eligible for the study or not having returned the questionnaires. Accordingly, for all the questionnaires, we have *n* = 40 for parent and *n* = 35 for adolescent. To test the first and second hypotheses, sensitivity and specificity scores were calculated to determine the predictive ability of the diagnostic tools used. The diagnostic power of a test needs to be determined by both the sensitivity and specificity. If the sensitivity is low, then true positives will be missed, but if the specificity is low, then there is little power to determine true negatives. How the sensitivity and specificity are assessed depends on the context of the testing scenario, and how important each of these is in relation to the requirements of the specific case (Baron, 1994; Boyko, 1994). Kappa statistics were calculated to determine convergence between scales. Landis and Koch (1977) specify the following interpretive guidelines: no agreement = below 0; slight = 0–0.20; fair = 0.21–0.40; moderate = 0.41–0.60; substantial = 0.61–0.80; almost perfect agreement = 0.81–1. To test the third hypothesis, discriminant functional analysis can be used to determine the best combination of predictors for a given binary outcome (Lewis and White, 2009; Lix and Sajobi, 2010). Cronbach alpha scores were calculated to determine scale reliability, with scores over 0.7 preferred (DeVellis, 2011).

## RESULTS

### DEMOGRAPHICS

Education level ranged from having completed year 6–12 of schooling, with a median of year 9. The adolescent’s primary parent was female in 88% of cases and male in 12% of cases, which did not permit analysis of mother vs. father reporting. The parent’s age range was 33–57 years old, with an average age of 47 years (SD = 5.58). 18 parents (47.4%) were married or in a de facto relationships, and 18 (47.4%) were divorced/separated or single parents. Two parents (5.3%) were divorced or separated and had re-partnered with the adolescent’s current step-parent. The mean yearly income for the family as a whole was the $50,000–80,000 per year income bracket, with 17 families (45.9%) reporting earnings of less than $50,000 per annum; seven of these families reported an annual income of less than $20,000 per year. 11 of the parents (27.5%) had completed up to year 10 of secondary school. Five parents (12.5%) had completed year 12, nine (22.5%) had completed a TAFE diploma or certificate, 10 (25%) had completed a university bachelor degree, and 5 (12.5%) had completed a postgraduate university degree.

### SENSITIVITY AND SPECIFICITY OF MEASURES OF ADOLESCENT MAJOR DEPRESSIVE EPISODES

First, we examined the sensitivity and specificity of measures with respect to the parent KID-SCID as the “gold standard” or reference point as shown in **Table 1**. We found that the adolescent’s KID-SCID-MDE showed high sensitivity (0.86) but only moderate specificity (0.38). Alternatively, when the adolescent’s KID-SCID was taken as the “gold standard,” the sensitivity for the parent proxy account based on the KID-SCID of their child’s depressive symptoms was much lower at 0.66, but the specificity was improved at 0.67.

**Table 1:** Diagnosis, convergence, sensitivity, and specificity scores using the Adolescent KID-SCID MDE module or the parent KID-SCID MDE module.

	Diagnosis	Adolescent KID-SCID			Parent KID-SCID		
	*n*(%)	Conv.	Sensitivity	Specificity	Conv.	Sensitivity	Specificity
**Adolescent**
KID-SCID	38 (76.0)	–	–	–	0.26	0.86	0.38
SMFQ	29 (82.9)	0.34	0.89	0.43	0.17	0.77	0.08
SDQ-ES	27 (77.1)	0.41	0.86	0.57	0.13	0.73	0.15
**Parent**
KID-SCID	29 (58.0)	0.26	0.66	0.67	–	–	–
SMFQ	33 (82.5)	0.13	0.85	0.29	0.05	0.84	0.20
SDQ-ES	38 (95.0)	0.08	0.94	0.00	0.14	0.96	0.07

*Conv, convergence, kappa score SCID, KID-SCID MDE module; SMFQ, Short Mood and Feelings questionnaire; SDQ-ES, Strengths and Difficulties Questionnaire Emotional Symptoms Subscale.*

We then examined our three questionnaire measures of depressive symptoms in relation to the diagnosis of MDE by both the parent and adolescent diagnostic interviews. As presented in **Table 1**, the adolescent’s self-reported SMFQ better predicted the adolescent KID-SCID outcome than it predicted the parent’s KID-SCID outcome. The parent SMFQ also better predicted the adolescent KID-SCID as compared to the parent KID SCID. This suggests that the adolescent reported KID-SCID is more closely related to the SMFQ scales than the parent reported version of the KID-SCID. A similar trend is seen for the parent version of the SMFQ, as it also better predicted the adolescent KID-SCID compared with the parent KID-SCID, as shown in **Table 1**. Notably, when the adolescent self-reported via the SMFQ, it provided a good prediction of their own response to the SCID interview (sensitivity = 0.89, specificity = 0.43), while the parent report via the SMFQ predicted their own report of their adolescent’s symptoms via the KID-SCID but with reduced specificity (sensitivity = 0.85, specificity = 0.20).

The SDQ-ES scale performed well when completed by the adolescent but seemed to invite over-reporting by parents. The adolescent SDQ ES scale performed well when predicting the adolescent KID-SCID diagnosis with high sensitivity (0.86) and a much improved specificity of 0.57. The adolescent version predicted the parent KID-SCID less well, as shown in **Table 1**. The parent SDQ ES scale diagnosed 38 cases out of 40 cases, and hence the very high sensitivity (0.94 and 0.96), and very low specificity of the scale (0 and 0.07, respectively). This suggests that parents over-reported depressive symptoms using the SDQ when compared to results derived from either the adolescent or parent KID-SCIDs. Convergence calculations indicate that the adolescent measures when predicting adolescent KID-SCID outcomes had the highest convergence, with the results classified as poor.

### SENSITIVITY AND SPECIFICITY OF MEASURES OF ADOLESCENT SUICIDALITY

We then examined the performance of our variance measures in relation to the adolescent’s report of suicidal ideas and behavior; which were self-reported by two thirds of our sample. These findings are reported in **Table 2**. When the adolescent’s response to the suicide item on the KID-SCID was taken as the “gold standard” or reference point, the sensitivity for the parent’s response to this same item was only moderate at 0.57, but the specificity was high at 0.82. Notably only 40% of parents considered their child to be suicidal and, assuming the adolescent self-reports were accurate, this suggests that parents may under-report, or be less aware of their adolescent’s suicidal thoughts and behavior. When the parent response of the suicide item was used as the “gold standard,” the adolescent showed a higher sensitivity (0.87) and only a moderate specificity (0.47).

**Table 2:** Diagnosis, convergence, sensitivity, and specificity scores using the Adolescent KID-SCID suicidality item or the parent KID-SCID suicidality item.

	Diagnosis of suicidality	Adolescent			Parent		
	*n*(%)	Conv.	Sensitivity	Specificity	Conv.	Sensitivity	Specificity
**Adolescent**
KID-SCID-SI	23 (67.6)	–	–	–	0.32	0.87	0.47
SMFQ	26 (89.7)	0.07	0.91	0.14	0.14	0.00	0.83
SDQ-EM	25 (86.2)	0.01	0.86	0.14	0.14	0.27	0.92
**Parent**
SCID-SM	15 (44.1)	0.32	0.57	0.82	–	–	–
SMFQ	29 (85.3)	0.06	0.84	0.11	0.01	0.86	0.15
SDQ-ES	32 (94.1)	0.10	0.96	0.11	0.02	0.93	0.05

*Conv, convergence, kappa score KID-SCID-SI, KID-SCID suicidality item; SMFQ, Short Mood and Feelings questionnaire; SDQ-EM, Strengths and Difficulties Questionnaire Emotional Symptoms Subscale.*

Overall, the sensitivity of the questionnaires on depressive symptoms in relation to the adolescent and parent suicidality items were high, ranging from 0.83 to 0.96. The specificity scores, however, were very low, ranging from 0.05 to 0.17 (with the exception of the adolescent SDQ). This indicates that the depressive symptoms questionnaires do not perform well in correctly identifying cases where the adolescent is not suicidal. While it is often the case that high depressive symptoms are accompanied by suicidal thoughts and sometimes behaviors, our findings here suggest that depressive symptom scales are likely to be over-inclusive if thought of as good proxies to identify an adolescent in danger of suicide. Direct questions to adolescent on their own suicidality perform with greater accuracy and thus appear to be preferable. Likewise, convergence statistics indicated poor convergence between the adolescent and parent KID-SCID suicide items and the diagnostic scales.

### DISCRIMINANT FUNCTION ANALYSIS

To determine the extent to which the Parent SCID plus the questionnaire data (SMFQ adolescent and parent, SDQ-ES adolescent and parent and Adolescent DEQ-A) could be used to identify adolescents who were diagnosed as having a MDE on the Adolescent SCID (diagnosis, no diagnosis), these variables were entered into a discriminant function analysis. When the variables were entered together, results revealed a function that correctly classified 92.9% of adolescents with a diagnosis, and 85.7% of the adolescents with no diagnosis. The overall classification was 91.4% [χ^2^(7) = 17.31, *p* = 0.016, Wilk’s lambda = 0.56, canonical *r* = 0.67] which accounts for 44.9% of variance in the model. Coefficients, standardized discriminant function and structure coefficient for diagnosis and no diagnosis groups on the adolescent SCID are shown in **Table 3**.

**Table 3:** Standardized discriminant function and structure coefficient for diagnosis and no diagnosis groups on the Adolescent KID-SCID, major depressive episode.

	Coefficient	*r*_s_	*r*_s_^2^(%)
**Parent**
Parent KID SCID	-0.086	-0.066	0.45
SDQ (ES) parent	0.775	0.096	0.92
SMFQ parent	0.330	-0.172	2.89
**Adolescent**
SDQ (ES) adolescent	0.484	0.501	25.10
DEQ-A efficacy	0.080	0.007	0.01
DEQ-A dependency	0.973	0.695	48.30
SMFQ adolescent	-0.142	-0.406	16.48

*Coefficient refers to standardized coefficient; *r*_s_, structure coefficient; *r*_s_^2^, square of structure coefficient as a measure of unique variance accounted for by this variable.*

In order to test the most parsimonious model, the scales that contributed most to the full analysis [SMFQ Adolescent, SDQ (ES) Adolescent, DEQ-A Dependency], which were all adolescent scales, were tested as a discrete model. When these three adolescent variables were entered together, the resulting discriminant function correctly classified 85.7% of the adolescents with a diagnosis, and 71.4% adolescents with no diagnosis, with the same overall classification of 82.9% [χ^2^ (3) = 12.06, *p* = 0.007, Wilk’s lambda = 0.68, canonical *r* = 0.56). This accounted for 31.3% of the variance in this model.

## DISCUSSION

In this paper we considered whether the use of parent proxy reporting and interview measures adds to the evaluation of adolescent depression. Parent proxy reporting is often assumed to make an important contribution to evaluation studies of adolescent mental health and is recommended in clinical guidelines (AACAP, 2007). In line with our first hypothesis we found that adolescent self-reports and parent reports of adolescent symptoms showed low concordance. In line with our second hypothesis the adolescent self-reports were better predictors than parent reports of the adolescent’s mental state as identified via a clinical interview using the KID-SCID MDE. The adolescent self-report was consistently superior to the parent’s report of the adolescent, even when parents used a parent version of the KID-SCID. The parent proxy questionnaires better predicted the adolescent’s KID-SCID MDE outcomes than the parent proxy KID-SCID.

The adolescent questionnaire diagnostic scales have high sensitivity and moderate specificity when predicting adolescent KID-SCID MDE outcomes. The parent questionnaire diagnostic scales had high sensitivity but lower specificity. The adolescent-report questionnaires more accurately reported adolescent depression than the parent report questionnaires. We also found that when parents used the Strength and Difficulties questionnaire to rate depression within a clinical sample, they tend to overstate the degree and severity of their adolescent’s depressive symptoms.

With regard to the assessment of suicidality, both adolescent and parent questionnaires examining depressive symptoms have very low specificity in identifying suicidality. We also found that, when compared to the adolescent’s self rating of their suicidality, where two thirds meet the DSM criteria, less than half of the parents agreed that their children were suicidal. This is a particularly important finding since it suggests that parents may underreport adolescent suicidal thoughts and behavior and that some parents are either poor judges or not aware of that their adolescents are suicidal.

For a clinical trial, it is particularly important that the measures used have a high degree of sensitivity so that participants who meet diagnostic criteria are appropriately identified. This is the most important aspect of classification in a clinical trial since it speaks to the validity of the trial’s claim to treat the target disorder. A lower specificity score will result in a participant being included in a trial but may not meet full diagnostic criteria and this would impact on the ability of the trial to determine intervention efficacy.

Our findings are consistent with previous studies that have found that parent reports of an adolescent’s subjective experiences such as depressive symptoms are less accurate (Grills and Ollendick, 2002). Potential resolutions to the conundrum of parent-child discordance need to be considered in terms of the context in which information is collected. For many clinicians, collecting both parent and child/adolescent information remains an important component of accurate assessment. However, it should be noted that even in a clinical context, parent reporting can be inaccurate and should not be used as a valid substitute for information gathered directly from the adolescent. From a clinical perspective, the current findings that parents may under-report suicidal thoughts and behavior are particularly notable. Lewinsohn et al. (1996) noted that while depression and suicidality are strongly correlated, that suicidal ideation is a unique predictor of future suicide attempts and should therefore be routinely assessed in addition to depression. Our findings that parents are poor reporters of adolescent suicide is important to consider when developing assessment guidelines for clinical assessment of suicide risk in adolescents.

In line with our third hypothesis, discriminant functional analysis also showed that parent reporting performed poorly whereas adolescent reported data performed very well in predicting adolescent diagnosis on the KID SCID. The optimal combination of measures for classification emerged as adolescent reported SDQ (ES), DEQ-A Dependency and SMFQ which showed a high degree of classificatory accuracy in the ability to discern the presence and absence of diagnosis. These findings are inconsistent with current clinical guidelines on the assessment of adolescent depression but consistent with numerous previous findings of poor concordance of parent and youth reports. Of high significance for research designs, an approach assessing adolescents with both questionnaire and structured interview methods for classification proved to be more effective. These findings for adolescent depression are distinct from other recommendations for multi-informant assessment of other mental disorders. For example, Thompson recently reported that the combination of parent and adolescent report using the “atypicality” scale of the Behavior Assessment System for Children, 2nd Edition (BASC-2) significantly improved prediction of structured interview for psychosis-risk syndromes (SIPS) over either single-informant scale. The study concluded that a multi-informant approach may help identify adolescents at risk for psychosis, particularly if used in conjunction with adolescent self-report (Thompson et al., 2014).

Within a research context, the degree of discordance identified in the current study is concerning. Accurate assessment of inclusion criteria and evaluation of clinically significant improvement is reliant on accurate classification. One of the major practical considerations in running a clinical trial is the degree of participant burden imposed with lengthy measures, and this has been reported to be particularly troublesome for adolescents (Lewis et al., 2012a).

There are a number of limitations within the current study that should be noted. In addition to the small sample size, the data is derived from a clinical study where adolescents suspected of having depressive disorders are referred for treatment. As such the findings may not generalize to less specific clinical settings. Other modes of assessing concordance could have been undertaken. Interestingly, Lauth et al. (2010) reported that item level analysis of both diagnostic and self reported measures may produce higher reliability and possibly greater accuracy in assessing clinical change at a finer gradient. Our interest was in classification and psychiatric diagnosis so we have not undertaken such an analysis. Internal reliability for measures was generally acceptable with the exception of the parent report of SDQ-emotional subscale and some subscales of the DEQ-A which may require further psychometric evaluation in clinical samples. Our sample included predominately females and mostly mothers as the parent informant, so we were unable to examine parent and adolescent gender effects. As such, our results most likely reflect the concordance of mother to daughter ratings. Our sample is restricted to the age range of 12–18 years in order to focus on adolescent depression but there is scope for future work on younger children examining the role of multi-informant classification of depression and this would be possible with children as young as Pre-School age where there is evidence of early onset of depressive symptoms (Luby et al., 2004; Lewis and Olsson, 2011).

This study provides further evidence of discrepancies between parent and adolescent reporting, with particular relevance to the clinical and research assessment of adolescent depression and suicidality. The parent proxy KID-SCID has low concordance with the adolescent’s clinical interview and is not as good a predictor of adolescent depression. Parent proxy report is less reliable but in a context where an adolescent refuses to provide information, it could be used to provide clinically relevant information. Parent report of adolescent depression and suicidal thoughts and intensions is of a lesser quality than that of the adolescent’s own report of the symptoms and subjective experiences. These are significant findings to consider within standard clinical assessment as well as within clinical research trials.

## Conflict of Interest Statement

The authors declare that the research was conducted in the absence of any commercial or financial relationships that could be construed as a potential conflict of interest.
